# Analysis of determinants of postpartum emotional disorders

**DOI:** 10.1186/s12884-021-03983-3

**Published:** 2021-07-20

**Authors:** Grażyna Iwanowicz-Palus, Agnieszka Marcewicz, Agnieszka Bień

**Affiliations:** Chair and Department of Development in Midwifery, Faculty of Health Sciences, Medical University of Lublin, Lublin, Poland

**Keywords:** Postpartum emotional disorders, Sense of coherence, Determinants

## Abstract

**Background:**

The birth of a child entails major changes in a woman’s life. In the perinatal period, the woman is particularly susceptible to emotional problems. The objective of the present paper was to investigate the relationship between global orientation to life and its components on the one hand, and socio-demographic factors on the other, with regard to early postpartum emotional disorders.

**Methods:**

The study included 643 patients hospitalized in obstetric departments in Lublin, Poland, who had had a spontaneous vaginal delivery. Research instruments included: the Edinburgh Postnatal Depression Scale (EPDS), the Orientation to Life Questionnaire (SOC-29), and the authors’ own survey questionnaire to record participants’ characteristics.

**Results:**

The study findings indicate an association between lower levels of postpartum blues and higher levels of global sense of coherence, as well as a stronger sense of meaningfulness, manageability, and comprehensibility. More severe emotional disorders were found in patients who were single. Postpartum blues symptoms were more intense in less educated respondents.

**Conclusions:**

Postpartum emotional disorders are associated with a global sense of coherence and its components. Higher levels of SOC reduce the risk of postpartum blues.

**Supplementary Information:**

The online version contains supplementary material available at 10.1186/s12884-021-03983-3.

## Background

The perinatal period is characterized by a number of concurrent biological, emotional and social changes, which require considerable personal and interpersonal adaptation on the part of the woman. During that time, the woman is particularly susceptible to a variety of psychological disorders [1, 2]. The birth of a child is a stressful event which causes major changes in a family’s life [3]. Based on the DSM III-R classification, the American Psychiatric Association classified the birth of one’s first child as a highly stressful event, assigning it to grade 4 out of 6 [4].

Postpartum blues is a transient, self-limited affective disorder. Its symptoms include irritability, mood swings, sleep disorders, attention disorders, and feelings of helplessness due to a perception of oneself as incompetent in one’s role as a mother. Postpartum blues is found in 30–80% of women in the first two weeks of the postpartum period [5]. Postpartum depression is a psychological disorder that shares its characteristics with depressive episodes occurring at any other life stage. According to the WHO, it develops in 10–20% of mothers, reaching peak severity between 2 and 6 months after giving birth [5]. The negative emotions are most commonly directed towards the newborn baby. Low mood, tearfulness, fatigue, loss of interest, and low self-esteem may occur as early as the second or third trimester of pregnancy, signaling the risk of postpartum depression. The negative consequences of perinatal depression may have a long-term impact on the mother’s bond with her child and her family, and may interfere with shaping the woman’s role as a mother [6, 7]. Therefore, prevention aiming at identifying women at risk of depression and those already affected is extremely important. In Poland, this need is addressed by the Ordinance of the Minister of Health of August 16, 2018, on perinatal care standards. This document was introduced to ensure a common, correct standard for medical personnel to follow regardless of the setting in which care is provided. It provides for proper prenatal preparation through quality education aimed at reducing the anxiety associated with labor and subsequent care for the newborn baby, and limiting the risk of depression. The Edinburgh Postnatal Depression Scale (EPDS) is the recommended research instrument for identifying either symptoms of postpartum mood disorders or risk of postpartum mood disorders [8]. So far, the etiology of postpartum depression has not been clearly identified. Global research indicates that the most common risk factors include: high-risk pregnancy, premature birth, hospitalization during pregnancy, high level of neuroticism, history of depression, and low level of social support [9].

Sense of coherence (SOC) or “global orientation to life” describes one’s way of seeing the world as predictable, manageable, and orderly. Research by Antonovsky indicates that SOC changes during one’s lifetime, affected by one’s life experiences. It also depends on an individual’s strength and ability to adapt effectively to stressors encountered in life [10].

SOC plays an important role in the maintenance of emotional health. Postpartum patients with a strong sense of coherence demonstrate positive, child-centered attitudes [11]. Those with a poor SOC are more likely to experience anxiety and mood disorders [12].

The objective of the present study was to investigate the relationship between global orientation to life and its components on the one hand, and socio-demographic factors on the other, with regard to early postpartum emotional disorders.

## Material

The study included 634 patients who had had a spontaneous vaginal delivery and were hospitalized in obstetric departments of university hospitals in Lublin, Poland, in the years 2015–2016. Women after a cesarean section take longer to recover than after a vaginal delivery, which means that more external factors may affect their psychological condition. Cesarean delivery may result in a sense of losing control over one’s situation and a lower self-esteem. Women who have had an emergency cesarean section may develop post-traumatic stress disorder. As physical contact with the newborn baby is limited after a cesarean delivery for medical reasons, the woman may have more difficulty in bonding with the baby, which has also been associated with poorer mood. Patients aged ≥ 18 years, who had had spontaneous vaginal delivery, were recruited on their 3rd or 4th postnatal day. Exclusion criteria were as follows: birth before week 38 of the pregnancy, the newborn requiring specialist treatment, or the diagnosis of depression, schizophrenia, or neurotic disorders before or during the current pregnancy. Patients were informed that participation in the study would be strictly voluntary and anonymous, and that results would only be used for scientific purposes. Data on patients’ physical and psychological condition were obtained by analysis of medical records and physical examination findings. Respondents were informed about the study purpose, signed the Informed consent form, and then completed the surveys themselves. Surveys were provided to patients and collected from them in person. Seven hundred 10 surveys were distributed; 634 correctly completed surveys were returned; 76 patients were not ultimately included in the study, as 31 failed to meet the inclusion criteria, 22 declined participation, 15 patients were found to meet the exclusion criteria (confirmed psychological disorders), and 8 children’s condition worsened so as to require specialist care. Surveys took approx. 15 min to complete, and the return rate was 89.29%. Each completed survey was reviewed. Patients with EPDS scores over 12 points or any self-harm intention were put in touch with a psychologist or psychiatrist to verify the test result, and undergo any follow-up testing for diagnosis. The authors obtained approval from the Bioethics Committee (approval no. KE-0254/56/2014) of the Lublin Medical University, written informed consent from all patients, and approval from all the health care institutions involved. 

## Methods

The study was performed using a diagnostic survey with questionnaires. We used standardized research instruments: the Edinburgh Postnatal Depression Scale (EPDS) and the Orientation to Life Questionnaire (SOC-29), as well as a original survey questionnaire.

The EPDS comprises 10 statements describing various aspects of a woman’s mood, including: anhedonia, guilt, anxiety, panic attacks, fatigue/overload, sleep disorders, sadness/depression, tearfulness, and suicidal ideation. The patient chooses one of the proposed responses to each statement. Scores for each statement range between 0 and 3 points. The total score is the sum of all item scores, with a maximum of 30 points. Higher scores indicate a higher probability of postpartum depression. Increased risk of depression is indicated by scores of 12–13 points or higher. In the overall evaluation, any positive score for suicidal ideation should be considered alarming, even with a low total EPDS score. The questionnaire’s reliability in the original study was measured by internal consistency (Cronbach’s α) is 0.88. Cronbach’s α for the EPDS in Polish settings is 0.91 [13].

The Orientation to Life Questionnaire (SOC-29) designed by Aaron Antonovsky measures the “sense of coherence” (SOC), also called “global orientation to life”. It reflects an individual’s perception of the world as predictable, manageable, and worthy of commitment. SOC has three components: the sense of comprehensibility — belief in the predictability and coherence of stimuli coming in from one’s external environment; manageability — belief in one’s ability to meet the requirements posed by these stimuli; and meaningfulness — an attitude of readiness to commit and make an effort. The questionnaire comprises 29 items in three subscales, reflecting the three components of coherence: manageability, meaningfulness, and comprehensibility. The respondent rates each statement in reference to themself and their life on a 7-item scale. The Cronbach’s α is 0.85 for the entire questionnaire, and between 0.72 and 0.75 for each subscale [10, 14]. The Polish version of the SOC-29 questionnaire has been found highly reliable. Internal consistency values were as follows: 0.92 for global sense of coherence, 0.78 for comprehensibility, 0.72 for manageability, and 0.68 for meaningfulness, while Cronbach’s α ranged between 0.82 and 0.95 in different studies [15].

Original survey questionnaire included sociodemographic variables like age, education, residence, relationship status, number of children, professional activity (Additional file 1).

### Statistical analysis

Statistical analyses of data from the questionnaires were performed using the SPSS Statistics 25.0 software. Correlations between socio-demographic factors, the incidence of early postpartum emotional disorders, and the global SOC and its components were calculated using the non-parametric Spearman’s rho correlation coefficient. Predictors of postpartum emotional disorders were identified using stepwise hierarchical regression analysis. This method involves stepwise inclusion in the model of variables with the most significant impact on the dependent variable, thus limiting the problem of variable correlation. Based on standardized regression residual values, 6% of outliers were removed. To find which variables, out of SOC components and socio-demographic factors, are significant predictors of emotional disorders in the postpartum patients, hierarchical regression analysis was used. In the first step, SOC was considered, and in the second, marital status and education were also included. The dependent variable was the severity of postpartum emotional disorders. Two models were built: one for SOC components, and another for global SOC. Findings at *p* < 0.05 were considered statistically significant.

## Results

Table 1 shows respondents’ characteristics. The most numerous age bracket was 31–35 years (30.1%). Most respondents declared having completed higher education (54.6%), lived in province capitals (38.0%) or other cities (38.0%), were married (81.1%), had two or more children (50.5%), and were professionally active (59.9%).

**Table 1:** Participants’ characteristics

Participants’ characteristics		n	%
Age	< 26 y/o	173	27.3
	26–30 y/o	169	26.7
	31–35 y/o	191	30.1
	> 35 y/0	101	15.9
Education	primary or vocational	110	17.3
	high school	178	28.1
	college/university	346	54.6
Residence	urban – province capital	241	38.0
	urban – other	241	38.0
	rural	152	24.0
Relationship status	single	120	18.9
	married	514	81.1
Number of children	one child	314	49.5
	two or more children	320	50.5
Professional activity	professionally active	380	59.9
	professionally inactive	254	40.1

Table 2 shows the descriptive statistics and Kolmogorov–Smirnov normality test results for postpartum depression, global SOC, and its components. Significant results of the test indicate that the distribution of the analyzed values is not normal.

**Table 2:** Mean scores for postpartum emotional disorders and global orientation to life in the women studied

Variables	**M**	**SD**	**Min**	**Max**	**Z**	***p***
Postpartum emotional disorders	8.18	5.40	0	22	0.10	0.001
Global sense of coherence	125.73	20.62	79	184	0.12	0.001
Comprehensibility	46.12	9.78	27	77	0.13	0.001
Manageability	45.57	7.80	24	64	0.07	0.05
Meaningfulness	34.05	6.35	19	44	0.10	0.001

*M* mean, *SD* standard deviation, *Min/Max* minimum/maximum value, *Z* Kolmogorov–Smirnov test statistic, *p* Z-test significance

Table 3 shows the associations between global SOC and its components on the one hand, and postpartum emotional disorders on the other. Statistically significant, moderately strong negative correlations were found between postpartum emotional disorder severity and global sense of coherence (*p* < 0.001), comprehensibility (*p* < 0.001), manageability (*p* < 0.001), and meaningfulness (*p* < 0.001). This indicates that more severe postpartum emotional disorders are associated with a lower level of global sense of coherence and its components.

**Table 3:** Correlation between global SOC and its components, and postpartum emotional disorders

SOC	Postpartum emotional disorders	*p*
Comprehensibility	-0.42	< 0.001
Manageability	-0.39	< 0.001
Meaningfulness	-0.41	< 0.001
Global sense of coherence	-0.46	< 0.001

Spearman’s *rho*

Statistically significant correlations were found between postpartum emotional disorder severity, and patients’ marital status (*p* < 0.05) and education (*p* < 0.05). More severe postpartum emotional disorders were associated with being single and with lower education levels (Table 4).

**Table 4:** Socio-demographic factors and postpartum emotional disorders

	Postpartum emotional disorders	*p*
Age	-0.03	> 0.05
Marital status^a^	-0.18	< 0.05
Education	-0.16	< 0.05
Residence	-0.02	> 0.05
Professional activity	0.02	> 0.05
Number of children	-0.08	> 0.05

^a^ Marital status: 0 — single; 1 — married. Spearman’s *rho*

Table 5 presents a summary of hierarchical regression coefficients for global SOC and its components and socio-demographic factors on the one hand, and postpartum emotional disorders on the other. The analysis demonstrated that global SOC was significantly associated with postpartum emotional disorders. A weaker global sense of coherence was a predictor of more severe postpartum emotional disorders (β = –0.54; *p* = 0.001). The model accounted for 54% of variance in the dependent variable. Another model for predicting postpartum emotional disorders included two SOC components: meaningfulness and comprehensibility. It accounted for 53% of variance in the dependent variable. More severe postpartum emotional disorders were predicted by a weaker sense of meaningfulness (β = –0.33, *p* = 0.001) and a weaker sense of comprehensibility (β = –0.26, *p* = 0.01). Socio-demographic factors were not found significant for the model.

**Table 5:** Regression analysis for postpartum emotional disorders

Independent variables	*B*	SE	*β*	*t*	*p*
Global sense of coherence	-0.13	0.02	-0.54	-7.66	0.001
	***R***^**2**^** = 0.54; *F*(1.140) = 58.64; *p* = 0.001**				
(Constant)	20.94	1.98		10.56	0.001
Meaningfulness	-0.38	0.06	-0.49	-6.71	0.001
	***R***^**2**^** = 0.49; *F*(1.141) = 44.95; *p* = 0.001**				
(Constant)	22.68	2.03		11.16	0.001
Meaningfulness	-0.26	0.07	-0.33	-3.58	0.001
Comprehensibility	-0.13	0.05	-0.26	-2.81	0.01
	***R***^**2**^** = 0.53; *F*(2.140) = 27.53; *p* = 0.001**				

Hierarchic stepwise regression analysis

## Discussion

Affective disorders are a widespread clinical problem among women in their postpartum period. The most commonly observed ones are postpartum blues and postpartum depression. According to researchers, the incidence of postpartum depression symptoms ranges between 30 and 75%, and varies depending on the research instruments used, study sample size, and study timing [16]. Niyonsenga et al. found a high level of postpartum depressive symptoms in 48% of the teenage mothers studied [17]. In a study by Tambaga et al. the prevalence of postpartum blues among women in the 2nd and 3rd month after giving birth was 34.5% [18]. The analysis by Edhborg showed that as many as 64% of women exhibited symptoms of postpartum blues in the first days after giving birth, but only 24% did after a week. Women with a history of postpartum depression were twice as likely to have subsequent depressive episodes in the 5-year follow-up period [19].

The results of the present study indicate a significant association between postpartum emotional disorders and global orientation to life. We confirmed that more severe postpartum emotional disorders are associated with a lower level of global sense of coherence. This is consistent with Antonovsky’s concept of salutogenesis [10]. These findings are also in line with those obtained by Sekizuki et al. who confirmed that a high level of SOC increases the effectiveness of coping with stress [20]. Results from a study by Ogawa suggest that women with low SOC scores were less resistant to stress, and their reactions to stress were more intense, increasing the risk of developing depression [21].

Significant predictors of postpartum emotional disorders in the present study included global SOC and two of its components: meaningfulness and comprehensibility. This finding corroborates Antonovsky’s concept, as the author attributed a particular role in shaping the sense of coherence to meaningfulness [22]. The sense of meaningfulness determines an individual’s motivation to act. A study by Rados et al. indicates that people with a strong sense of meaningfulness treat problems as challenges that are worth their effort and commitment [23]. Kurowska et al. observed that this group of people uses all their defense mechanisms to try and change their situation for the better [24].

In the present analysis, there was a notable association between postpartum emotional disorders and the women’s marital status. Many studies sought to identify the relationship between socio-demographic factors and postpartum depression. A study by Fei-Wan Ngai demonstrated that women in steady relationships have a high level of family-related sense of coherence, which helps them adapt to new life situations [25]. In turn, Charline El-Hachem did not observe any significant impact of relationship status on the prevalence of postpartum depression among single women [26]. Małus et al. reported that women who were more satisfied with their relationships were characterized by better psychological condition after giving birth [27]. Umuziga et al. found more severe symptoms of mood disorders after giving birth among respondents having four or more children, who had a poor relationship with their partner, compared to those who only had one child [28]. An association between postpartum depression symptoms and marital conflict was also observed by Brackington. One may suppose that the sense of security provided by a stable relationship is a precondition for a high quality of life [29].

The present study suggests that better self-esteem due to one’s higher educational level may have a protective effect against emotional disorders. Postpartum blues symptoms were higher in intensity in less educated respondents. A similar finding was reported by Davis et al. [30]. These observations may be explained by the relationship between education and household income, which in turn affects women’s concerns about raising a child. Contrasting data were reported by Vaezi et al. and Maliszewska et al. who found no association between postpartum depression and education [31, 32].

One major limitation of the present study is the inclusion of hospitalized patients only in the first postpartum days, which prevented generalization to the entire population of mothers. In subsequent studies, a longitudinal design should be applied to provide more complete information on postpartum depression.

Though the correlations between socio-demographic factors and postpartum depression observed here are weak and differ from other authors’ findings, they may become the starting point for further research, ultimately leading to a clearer understanding of factors predicting postnatal emotional disorders.

## Conclusions

Factors affecting postpartum emotional disorders include the global sense of coherence and its components, namely comprehensibility and meaningfulness, as well as socio-demographic variables — marital status and education. Higher levels of SOC reduce the risk of postpartum blues.

## Supplementary Information

**Additional file 1.**

## Data Availability

The datasets generated and / or analyzed in the current study are not available due to the end of operation of the company that analyzed our data and we lost access to our database.

## Abbreviations

EPDS: Edinburgh Postnatal Depression Scale
SOC: Sense of coherence
SOC-29: The Orientation to Life Questionnaire
WHO: World Health Organization
