# Supplementary material for: Analysis of determinants of postpartum emotional disorders
Source: BMC Pregnancy Childbirth. 2021 Jul 20;21:517. doi: 10.1186/s12884-021-03983-3 (PMC8293488; doi:10.1186/s12884-021-03983-3)

**Original survey questionnaire**

1. Age:
2. <26 years
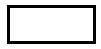

3. 26-30 years
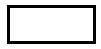

4. 31-35 years
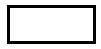

5. >35 years
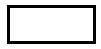

6. Education:
7. primary or vocational
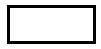

8. high school
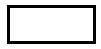

9. college/ university
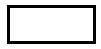

10. Residence:
11. urban- province capital
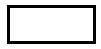

12. urban- other
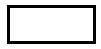

13. rural
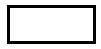

14. Relationship status:
15. single
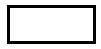

16. married
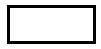

17. Number of children:
18. one child
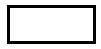

19. two or more children
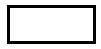

20. Professional activity:
21. professionally active
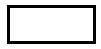

22. professionally inactive
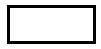

Supplement: Supplementary file 1 — Additional file 1. [file 12884_2021_3983_MOESM1_ESM.docx]
